# Degradation Characteristics and Service Life Prediction of Desert Sand Concrete Under Load and Freeze–Thaw Conditions

**DOI:** 10.3390/ma18215035

**Published:** 2025-11-05

**Authors:** Zhengyang Xia, Yongjun Qin, Ling Luo

**Affiliations:** School of Civil Engineering and Architecture, Xinjiang University, Urumqi 830047, China; 13581365408@163.com (Z.X.); luoling128@126.com (L.L.)

**Keywords:** desert sand concrete, sustained load, freeze–thaw cycles, frost resistance, service life prediction

## Abstract

Concrete structures in western China often endure severe freeze–thaw cycles under sustained loading. However, the combined effects of desert sand admixtures and long-term stress on freeze–thaw durability are insufficiently investigated. The existing research has focused on the material modification of desert sand concrete (DSC) or on the mechanical-environment coupling of ordinary concrete. This leaves a knowledge gap about how sustained compressive stress influences the macro- and mesoscale freeze–thaw behaviour of DSC. This study systematically investigated the freeze–thaw resistance of DSC under varying sustained compressive stresses. Testing methods and conditions were tailored to the climatic characteristics of China’s high-altitude cold regions. Freeze–thaw degradation was assessed using mass loss, relative dynamic modulus of elasticity, and compressive strength. Controlled loading effectively mitigated freeze damage. After cyclic freeze–thaw, the 0.3 and 0.5 stress groups retained 89.36% and 77.92% of their original compressive strength, respectively. Scanning electron microscopy, mercury porosimetry, and CT scanning revealed mesoscale damage mechanisms. Sustained loading optimized pore structure and enhanced compactness. A two-parameter Weibull probability model was then established to describe damage evolution patterns and assess the service life of desert sand concrete under regional climatic conditions.

## 1. Introduction

The rapid advancement of the worldwide construction sector has caused a notable rise in the use of natural sand and gravel resources in recent years, culminating in a scarcity of these materials [1]. Deserts are extensively scattered globally, including around 3140 km^2^, constituting 21% of Earth’s surface area; nonetheless, their use remains minimal. In regions abundant in desert sand resources, using desert sand (DS) efficiently in railway, highway, and related infrastructure projects enables the use of local materials. It effectively alleviates the current shortage of river sand resources [2,3]. It plays a crucial role in desertification control and ecological conservation [4,5], achieving the dual objectives of desertification prevention and promoting sustainable development. DS belongs to extra fine sand, which has poor grading in terms of particle size composition and does not meet concrete configuration standards [6]. The addition of a large amount of DS can slightly reduce the ease of use and compressive strength of concrete. Nevertheless, a moderate amount of DS is considered to be beneficial to optimizing the particle size distribution of fine aggregates, which improves the mechanical properties and durability of concrete [7,8,9,10]. Yan et al. [11] investigated desert sand concrete (DSC) proportioning based on a statistical modelling design and optimization method and proposed optimization guidelines for DSC mix ratio design parameters suitable for different construction requirements. Through reasonable mix ratio design, the compatibility and mechanical properties of DSC can meet the requirements of concrete structures.

The endurance of concrete, a crucial building material, has garnered significant scholarly study [12,13,14], with freeze–thaw cycling being a key aspect in assessing its durability [15,16]. Concrete exposed to freeze–thaw cycles (FTC) results in the formation of ice crystals inside its internal pores, which apply pressure on the pore walls of the matrix, hence facilitating fracture propagation [17,18]. Researchers [19,20,21] investigated the freeze resistance of DSC after incorporating different types of whiskers, elucidating the development process of its durability across various scales. Li et al. [22] investigated the freeze–thaw damage and degradation of DSC from a multi-scale approach, integrating both macroscopic and microscopic views. They posited that the incorporation of DS altered the internal porosity and pore architecture of the concrete, modifying the water transport pathway, which subsequently influenced pore saturation and saturation rate, decreased permeability, and improved frost resistance and durability.

Most concrete structures in southern Xinjiang, China, such as road surfaces, bridges, and buildings, are typically subjected to the combined effects of frost heave and external loads under cold climatic conditions. This composite degradation effect leads to severe durability degradation problems, manifested as accelerated concrete surface spalling and crack network expansion, significantly shortening the engineering structures’ service life. It is worthwhile to consider that the load freeze–thaw coupling will significantly change the mechanism of the evolution of microscopic damage in concrete material [23]. In the freeze–thaw damage process, the volume expansion caused by the phase shift in pore water and the stress field produced by external loading are combined, which jointly regulate dynamic evolution of the internal micro- and microscopic defects in the material [24]. Concerning the regulation mechanism of pressure level on frost resistance, experimental studies revealed a dual mechanism of action [25]: when lower compressive stresses (<30% of the ultimate compressive strength) are applied, the moderate confining effect of the load can optimize the distribution of the pore structure and inhibit the expansion stresses induced by the growth of ice crystals, which improves the durability of the material against freezing by about 15–20%. However, when the compressive stress level exceeds the critical threshold (>50% of ultimate compressive strength), the loading effect will trigger the nucleation and expansion behaviour of microcracks. The stress-induced microdefect network facilitates rapid water infiltration and exacerbates the freeze–thaw damage process via stress concentration at the crack tip, resulting in a nonlinear decline in the macroscopic mechanical properties of the material [26,27].

The Weibull distribution model constructed according to the probabilistic statistical theory has broad applicability in engineering reliability analysis [28], and its mathematical characterization properties significantly correlate with the damage evolution laws and failure modes of concrete materials. The theory can effectively characterize the random features of damage evolution induced by internal structural heterogeneity of brittle materials by establishing the probability density function of random variables [29]. It is widely used in the service life prediction study of concrete. Vu et al. [30] comprehensively investigated the performance of three different methods—the graphical method, the maximum likelihood method, and the moment method—for estimating the Weibull distribution parameters of concrete compressive strength. The effectiveness of these methods was compared using Kolmogorov–Smirnov and Anderson–Darling tests, as well as AIC and BIC methods. Wang’s group [31] systematically compared the reliability differences between regular concrete (NC) and polyethylene fibre-reinforced cementitious composites (PE-ECC) under freeze–thaw cycling conditions by establishing the damage dynamics equation considering the environmental coupling effect, combined with the Weibull probabilistic reliability curve. The study shows that the reliability model based on the three-parameter Weibull distribution has an error rate of less than 8% between the prediction results and the accelerated freeze–thaw test data, demonstrating good engineering applicability. The above research results validate the Weibull probabilistic statistical method’s theoretical value in durability assessment and life prediction of concrete materials, especially in dealing with asymmetrically distributed, small-sample failure data, which shows unique advantages.

This study investigates the damage mechanism of DSC under the coupled effects of continuous compressive loading and freeze–thaw cycles (FTC) in the high-altitude, cold regions of western China. Quantitative assessments of DSC’s freeze–thaw resistance were conducted using methods such as mass loss, relative dynamic elastic modulus (RDME), and compression strength loss. The microstructural characteristics of DSC were analyzed using scanning electron microscopy (SEM) and mercury intrusion porosimetry (MIP). This study provides a theoretical basis for the optimized design of DSC in terms of freeze–thaw durability, which is beneficial for the research and development of environmentally friendly building materials and their engineering applications.

## 2. Materials and Methods

### 2.1. Raw Material

**Figure 1:**
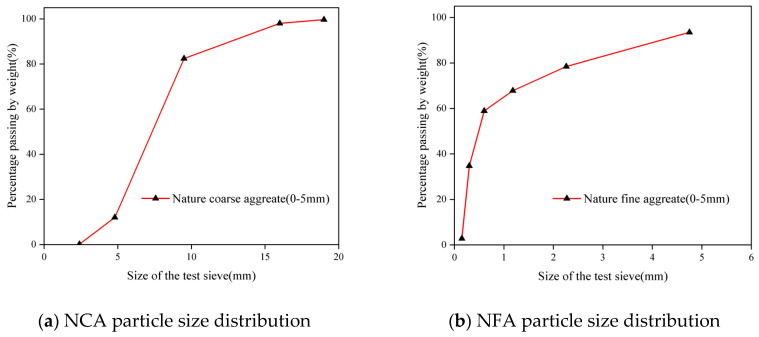
Particle size distribution of aggregates.

Natural coarse aggregate (NCA) was obtained from natural pebbles in China’s Xinjiang region with continuous grading of 5~20 mm in diameter. The natural fine aggregate (NFA) is washed medium-coarse sand from the Xinjiang region, with a fineness modulus of 2.54 and a water content of 3.73%. The particle size distribution of the aggregate is shown in Figure 1; the DS is taken from the Taklamakan Desert, with a fineness modulus of 0.122, an average grain size of 0.118 mm, a bulk density of 1334 kg/m^3^, an apparent density of 2790 kg/m^3^, and a water content of 0.4%, and the chemical compositions are tabulated in Table 1. The distributions of their grain sizes are shown in Table 2. The cement employed (P.O. 42.5, China Construction Western Engineering Co., Ltd., Xinjiang, Ürümqi, China) possessed a specific surface area of 350 m^2^/kg. Fly ash (China Construction Western Engineering Co., Ltd.) exhibited a specific surface area of 330 m^2^/kg. Granulated blast furnace slag powder (Henan Dingnuo Purification Materials Co., Ltd., Gongyi, Henan, China) comprised finely ground granulated blast furnace slag with a specific surface area of 430 m^2^/kg. Table 3 lists the principal oxide constituents detected in the three cement materials via Rigaku X-ray fluorescence (XRF) analysis in Tokyo, Japan. Tap water was employed for the experiments; the water-reducing agent (Sichuan Dongrun Baisheng New Materials Co., Ltd., Chengdu, Sichuan, China) was dosed at 4.47 g per cubic metre.

### 2.2. Design of Mix Ratio

Concrete of strength class C30 was employed in this study. The proportion design refers to the national standard JGJ 55-2011 [32]. It was further based upon the optimal mix design research outcomes obtained by this research group through the response surface method in prior studies [33]. Fly ash was used to replace 20% of the cementitious materials, DS replaced 30% of the fine aggregate, and the amount of steel slag powder was 10%. The water/cement ratio was 0.45. The DSC proportions are shown in Table 4.

### 2.3. Experimental Design

This study, in accordance with the ‘Standard Test Methods for Long-Term Performance and Durability of Concrete’ and its requirements for the ‘[34] Rapid Freezing Method’ test, selected two specimens of differing geometries for comparative analysis: a prismatic specimen measuring 100 mm × 100 mm × 400 mm, and a standard cube specimen with a side length of 100 mm. Among these, there are 3 sets of prismatic specimens and 21 sets of cubic specimens, with 3 specimens in each set, totaling 72 specimens. The experimental design mainly investigated the effects of key variables such as continuous load levels and the number of FTC on the material properties of DSC. In the loading parameter setting, the stress levels were divided into three gradients based on the proportionality of the axial compressive strength (*f*_c_) of the specimens: the high-stress level (H) corresponded to 0.5 *f*_c_, the medium-stress level (M) corresponded to 0.3 *f*_c_, and the zero-stress level (N) corresponded to 0 *f*_c_, to construct a multi-stress hierarchy of the test comparison system [24].

**Figure 2:**
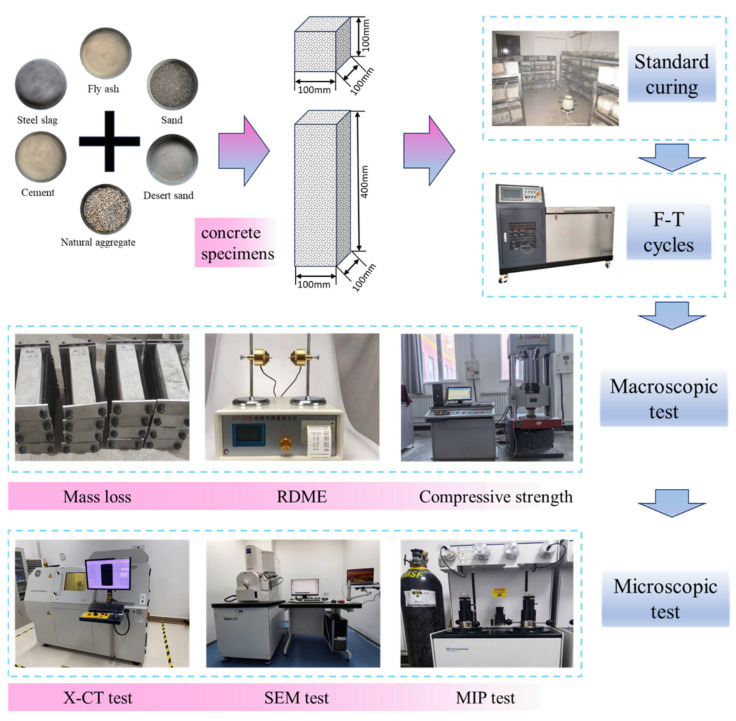
Schematic diagram of the production process of concrete specimens and the equipment required for their experimentation.

Figure 2 depicts a schematic representation of concrete specimen fabrication and outlines the laboratory apparatus used. Initially, the material was placed in a twin-shaft mixer and stirred then poured into a 100 mm × 100 mm × 100 mm cube mould and a 100 mm × 100 mm × 400 mm prism mould and vibrated to flatten it to make a test sample. After moulding, the specimen shall be immediately covered with an impermeable film. Following a 24 h rest period at 20 ± 5 °C, it shall be removed and transferred to a standard curing chamber for 24 days of curing (temperature 20 ± 2 °C, humidity ≥ 95%). Subsequently, freeze–thaw cycling tests (FTC) shall be conducted in a freeze–thaw testing machine.

### 2.4. Test Methods

#### 2.4.1. Application of Continuous Loads

**Figure 3:**
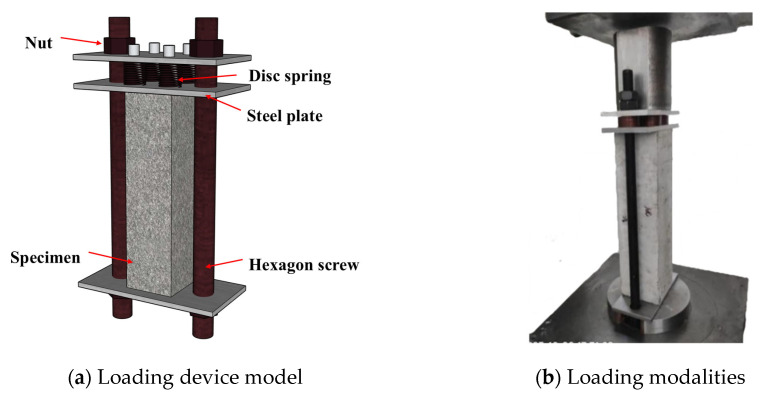
Continuously pressurized load application device.

Figure 3 illustrates the continuous loading apparatus and the loading procedure. The structure of the loading device includes three steel plates at the top, middle, and bottom, two bolt rods, butterfly springs, and customized nuts. Round holes were left at the ends of the loading plates to facilitate the later passage of the bolt rods, and a 100 mm × 100 mm scale was drawn in the middle of the bottom loading plate to determine the position of the concrete test block.

#### 2.4.2. F-T Cycle Test

Freeze–thaw testing of loaded specimens was conducted using the ‘rapid freezing method’ specified in GB/T 50082-2024 [34]. Samples underwent standard curing for 24 days, were then removed and placed in water at 20 ± 2 °C for 4 days, followed by freeze resistance testing. Prior to testing, surface moisture was removed, and initial mass and dynamic modulus of elasticity were measured. Each FTC was completed within 4 h, with the thawing phase constituting one-quarter of the total cycle duration. The core temperature of specimens was maintained at a minimum of −18 ± 2 °C and a maximum of 5 ± 2 °C. A single test cycle comprised 25 cycles per group.

#### 2.4.3. Mass Loss Test

Following multiple freeze–thaw cycles, surface spalling of the mortar reduces the concrete’s quality. Consequently, mass loss is commonly employed as a macroscopic indicator to evaluate freeze–thaw damage in concrete. After every 25 cycles of testing, mass loss assessment is conducted using prismatic specimens measuring 100 mm × 100 mm × 400 mm. Mass loss is calculated using Equation (1).

(1) $$\Delta M=(1-\frac{M_{n}}{M_{0}})\times 100\%$$

where Δ*M* denotes the mass loss rate, *M_n_* is the mass of concrete after undergoing *n* FTC, and *M*_0_ is the initial mass of concrete.

#### 2.4.4. Relative Dynamic Elastic Modulus Test

Following the specimen’s exposure to loading and FTC, the concrete specimen underwent destruction, resulting in the generation of numerous microcracks within its structure. The RDME can serve as a metric for evaluating the extent of internal damage to concrete. The lateral fundamental frequency can be calculated and determined in accordance with Equation (2):

(2) $$P_{n}=\frac{f_{n}^{2}}{f_{0}^{2}}\times 100\%$$

where *P_n_* (%) denotes the RDME after *n* FTC, calculated from the average of three specimens. *f_n_* (Hz) is the transverse fundamental frequency after *n* FTC. *f*_0_ (Hz) is the transverse fundamental frequency before the FTC test.

#### 2.4.5. Compressive Strength Test

The cubic compressive strength of the specimen is determined by Equation (3):

(3) $$f_{cc}=\frac{F}{A}$$

where *f_cc_* indicates the compressive strength of the concrete cube specimen (MPa); *F* indicates the specimen destructive load (N); *A* indicates the specimen compressive area (mm^2^). The cube compressive specimen used in this test is a non-standard piece, the size of 100 mm × 100 mm × 100 mm, considering the influence of the size effect, according to the calculation of the compressive strength, which needs to be multiplied by 0.95 coefficient.

#### 2.4.6. SEM Observation

In order to more intuitively analyze the damage deterioration mechanism of the DSC under the coupling of load FTC, SEM tests were conducted on specimen samples of DSC at high-stress levels (0.5 *f*_c_) for 0, 50, 100, and 150 times of FTC, respectively, to observe the change characteristics of the pore space, pores, and other microstructures within the DSC. The test samples were cut, soaked in anhydrous ethanol, ground, polished, and sprayed with gold, and later observed using field emission scanning electron microscopy.

#### 2.4.7. MIP Test

The mercury intrusion porosity method (AutoPore V 9620, Micromeritics, Norcross, GA, USA) was utilized to characterize the pore structure of DSC samples subjected to 0, 50, and 150 FTC. The principle underlying this method involves the application of external pressure to overcome the surface tension of mercury in the pores, thereby enabling the measurement of the intrusion volume of the mercury column. The intrusion volume of the mercury column can thus be quantified by measuring the intrusion volume versus the pressure change. In that case, the pore diameter (size) d can be related to the applied pressure P through the Washburn equation [35,36], see temporal Equation (4):

(4) $$d=-\frac{4\gamma \cos\theta }{P}$$

where γ is the surface tension of the mercury and θ is the contact angle between the mercury and the pore wall. Combined with this relationship, the pore size and its corresponding intrusion/extrusion volume can be determined.

Following the completion of the compression test, a sample measuring less than 5mm in edge length and weighing approximately 1–2 g shall be selected from the core region of the specimen. This sample shall be placed into a sampling bag and filled with anhydrous ethanol to prevent the occurrence of hydration reactions. The test sample is placed in the test container, degassed with a vacuum pump, and then the mercury is forced into the pores of the sample under pressure. Low-pressure tests were performed from 0 to 25 psi, followed by high-pressure tests from 25 to 60,000 psi.

#### 2.4.8. X-CT Analysis

Computed Tomography (CT) is an imaging modality that employs X-rays or γ-rays to irradiate an object and utilizes mathematical methods to generate a tomographic picture of the object. The scanned sample is a cube with a side length of 100 mm and an image resolution of 59.2 μm/pixel with an image pixel count of 1024 × 1024. CT scanning tests were performed on specimens of DSC subjected to 0, 50, and 150 FTC treatments under 0.5 *f*_c_ stress.

## 3. Results and Discussion

In this experiment, the effects of different sustained load levels and the incorporation of a certain number of DS, steel slag, and fly ash on the frost resistance of DSC were investigated by analyzing the macro-parameters such as mass loss, RDME, and compressive strength of the specimens. Representative DSC specimens in a 0.5 *f*_c_ stress state were selected and subjected to SEM testing, mercury intrusion testing, and CT scanning to observe changes in the internal pores, voids, and microstructure of DSC, thereby elucidating the mechanism of macro-performance degradation of DSC.

### 3.1. Mass Loss

**Figure 4:**
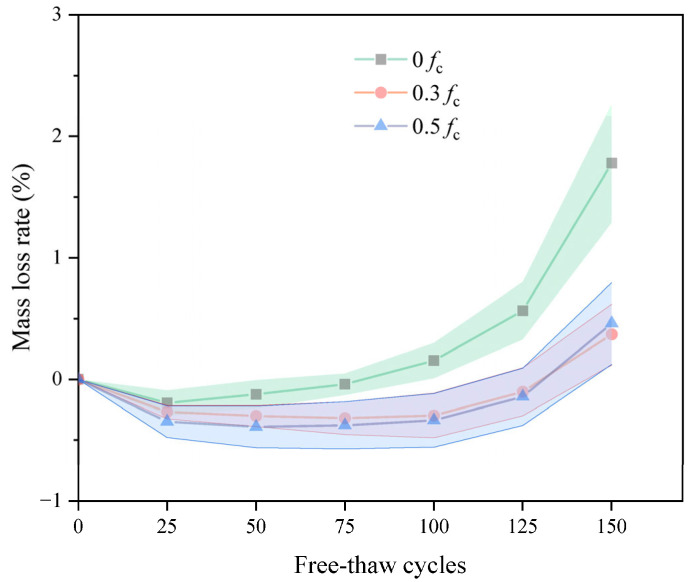
The mass loss versus FTC.

The mass loss rate of DSC is given in Figure 4. During the FTC, the mass loss of the samples at both medium- and high-stress levels was better than that of the unstressed group. This observation suggests that sustained compressive stress reduces the mass loss of concrete. During freeze–thaw cycling, although surface spalling reduces the mass of the specimens, the specimens likewise absorb water with the development of internal cracks, increasing total mass. As the FTC progresses, it is found that the mass loss of the medium-high pressure group is close. The mass loss of the medium-stress group was lower than that of the high-stress group in the early stage of the FTC. However, after 125 cycles, mass loss accelerated in the high-stress group and surpassed that of the medium-stress group. By the end of the freeze–thaw cycles, the high-stress and medium-stress groups had only decreased by 0.46% and 0.37%, respectively. This represented a lower mass loss of 1.41% and 1.32% compared to the group without pre-stressing. This is because, although applying a specific sustained load makes the specimen more compact, the excessive compressive stress causes the initially closed pores to crack after being subjected to compressive stress, resulting in connectivity, which aggravates the damage of the specimen.

### 3.2. Relative Dynamic Elastic Modulus

**Figure 5:**
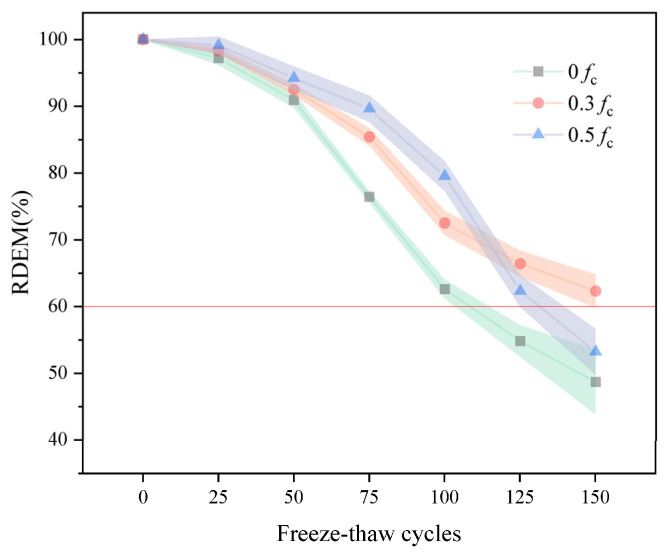
The RDME versus FTC (The red line indicates the damage threshold for freeze-thaw damage.).

Figure 5 displays the RDME values of desert sand concrete under various pressures. It may be shown that the RDME of the pre-loaded test block displays a steadily decreasing trend during the whole cycle. During 100 FTC, the continuous formation and growth of C-S-H gel, driven by ongoing cement hydration and the pozzolanic effect of mineral admixtures, resulted in no visible pore expansion occurring in the specimens during the initial stages of the FTC. It is worth mentioning that the RDME of the test block under high stress is more significant than that under medium load. This is because the concrete is not only harmed by the freeze–thaw impact during the test but also continues to hydrate in the freeze–thaw environment. More crucially, the internal pressure stress causes the internal structure of the concrete to become more compact, making it more difficult for water to enter the pores. Thus, it is less affected by FTC, and the formation of interior fractures is less severe in the early and middle phases of the FTC. It is worth noticing that in 100 to 150 cycles, the RDME of the test block under high stress steadily drops until around the 125th cycle, which is lower than that under medium stress. This indicates that while a certain pre-stress will make the specimen more compact in the early part of the FTC, the hydration reaction will achieve an equilibrium condition in the latter stage of damage. The damage produced by freeze–thaw and the damage induced by stress will operate on the test block simultaneously, causing the test block’s RDME to fall quickly, nearing failure. Fu et al. [24] similarly reported superior frost resistance for 0.3 fc over 0 and 0.5 fc in their RAC study on ‘Continuous Compressive Load–Freeze–thaw Coupling’. At 50 freeze–thaw cycles, the 50% recycled aggregate replacement rate combined with 0.3 fc stress group demonstrated the most favourable performance, achieving an RDME of 69.62%. Following 50 freeze–thaw cycles, all specimens exhibited RDME values below 60%. Comparing with the present study, a high degree of consistency was observed in the decline trend of relative dynamic modulus. However, the present study retained a greater relative dynamic modulus, with the unstressed group’s relative dynamic modulus decreasing to merely 62.6% by the conclusion of 100 freeze–thaw cycles. A possible explanation for this contrast may lie in the addition of desert sand, which significantly enhanced the concrete’s compactness.

### 3.3. Compressive Strength

**Figure 6:**
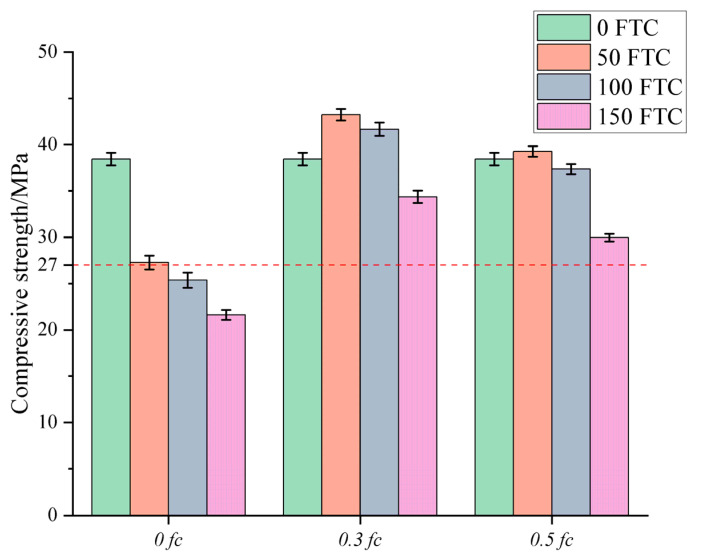
Results of the compressive strength test (The red line indicates the damage threshold for freeze-thaw damage).

Figure 6 shows the test results for the compressive strength of the DSC cube. The compressive strength of the unstressed group decreased significantly with the increase in the number of FTC, and the compressive strength of DSC decreased to 21.6 MPa after 150 FTC, which is a reduction of 43.82%. The loss rate of compressive strength was minimized in the specimens under medium stress conditions, and the specimens exhibited excellent performance, retaining 89.36% of the initial strength. Notably, the compressive strength of concrete in the first 100 FTC in the medium-high stress condition continued to increase, reinforcing that certain pre-stressing conditions will inhibit the development of cracks in the concrete, resulting in higher densification and better integrity. However, the specimens under high-stress conditions showed a greater loss in compressive strength than those under moderate-stress conditions. Although sustained loading enhanced the impermeability of the concrete and limited the development of microcracks, excessive sustained loading may exacerbate the damage. High-stress sustained loading disrupts the delicate balance between favourable and unfavourable factors during the FTC of concrete, resulting in progressively greater rates of loss in compressive strength. In their study on the ‘load-freeze–thaw coupling’ behaviour of C30/C50 concrete, Wang et al. [25] observed that water absorption and chloride ion permeability were lowest under the 0.3 fc loading condition, with permeability increasing sharply after exceeding 100 freeze–thaw cycles. This finding aligns with the lower mass loss, higher strength retention, and greater dynamic modulus retention observed in the 0.3 *f_c_* group within this study.

Consistent with the data for the RDEM, the compressive strength of the DSC showed increased variability after the FTC, indicating a high degree of change in its mechanical properties. This variability underscores the necessity for meticulous consideration of material property fluctuations when employing DSC in structural applications, particularly those necessitating resistance to freeze–thaw damage.

### 3.4. SEM Images

**Figure 7:**
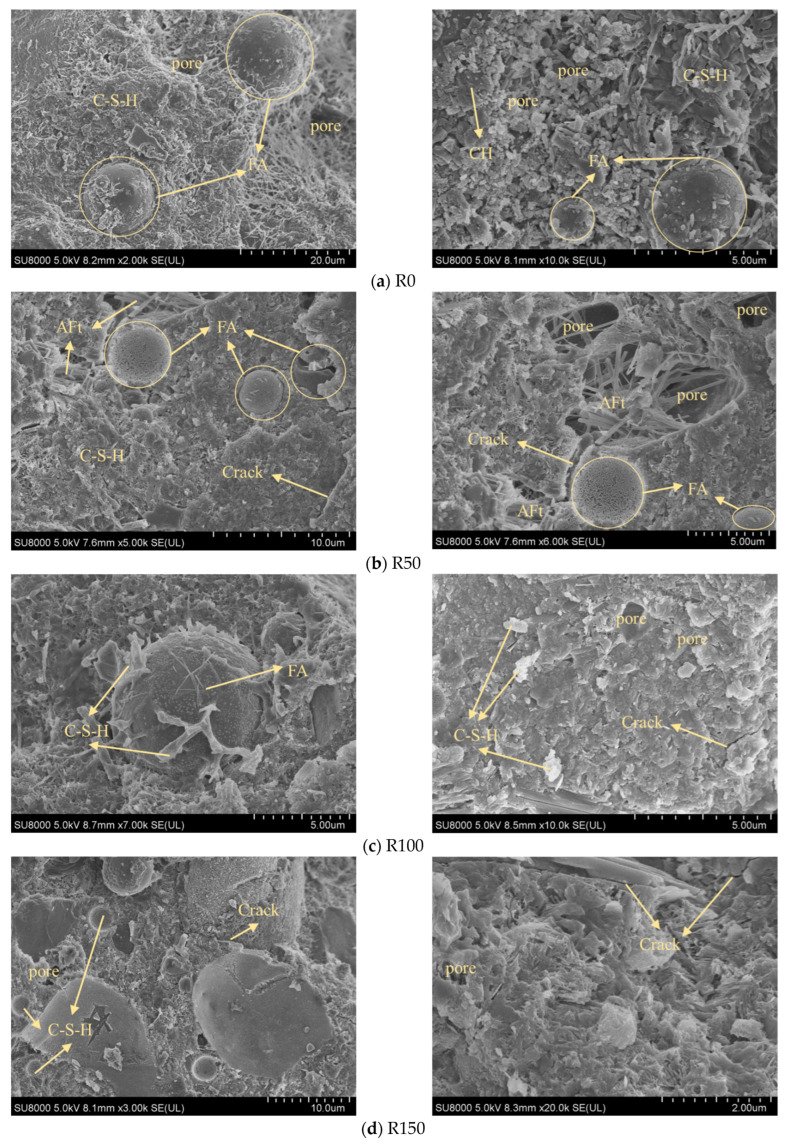
SEM images before and after freeze–thaw cycles.

Figure 7 shows the SEM images of the highly stressed DSC samples after different numbers of FTC. It was observed that with the hydration reaction in the early stages of FTC period, the hydration products combined and intertwined with each other and superimposed inside the DSC to form lamellar, laminar, and needle-and-rod structures, which filled the pores, stabilized the structure, and improved the strength of the concrete [37,38]. An adequate quantity of fly ash facilitates a secondary hydration reaction involving its substantial content of reactive SiO_2_, Al_2_O_3_, and Ca(OH)_2_ in the cement hydration products, producing hydrated calcium silicate (C-S-H) gel. This C-S-H obstructs the spatial development of the remaining hydration products, such as calcium hydroxide (CH) and calcite (AFt), resulting in the emergence of these two product types solely within the pore spaces and microcracks. These hydration products may penetrate the interior pores of the concrete, enhancing its integrity and density. Together with the external pressure factors, the crack expansion is extraordinarily slight and almost undetectable. Following 50 FTC, the quantity of hydrated products gradually increased, with gel coverage rising by 20%, while the gaps between these products correspondingly diminished. With the growth of time, the fly ash in the DSC was further hydrated, the surface of the fly ash became rough, and the obvious C-S-H gel could be found. It is noteworthy that the hydration time was significantly prolonged, and at the time of 100 times the FTC, the outer layer of the fly ash encapsulated the C-S-H gel. This indicates that the hydration process has been delayed due to the inhibition of steel slag on the precipitation of CH by lowering the pH in the pore solution. This results in the inhibition of nucleation and growth of C-S-H, thereby hindering the development of concrete strength [39]. The mechanism of steel slag involved in the reaction differs from that of fly ash, and a reaction analogous to cement hydration occurs upon the contact of steel slag with water. The mechanism of steel slag differs from the pozzolanic reaction of fly ash. Steel slag exhibits latent hydraulic properties, allowing it to react directly with water, albeit more slowly and to a lesser extent than Portland cement due to its crystalline structure. While direct hydration can form some C–S–H and Ca(OH)_2_, a substantial portion of the C–S–H gel in cement–steel slag systems originates from a secondary reaction. In this process, silicate and aluminate phases in the slag are activated by the Ca(OH)_2_ from cement hydration, yielding additional C–S–H and C-A-H [40,41]. The hydration of the main cementitious phase in steel slag does not react chemically with Ca(OH)_2_ produced by cement hydration, so the reaction of steel slag only contributes marginally to increasing Ca(OH)_2_ in the system [39], and the products of pre-hydration by steel slag are difficult to detect. Nonetheless, the applied pressures impeded the initiation and progression of fractures, demonstrating substantial compressive strength during the first and intermediate phases of the FTC [24].

As the FTC continues, the hydration reaction continues, and unreacted fly ash particles can still be observed. However, at this time, the internal damage to the concrete is more serious, and the hydration reaction has been ineffective in improving the compactness of the concrete. The number of pores in the concrete increases, and the pore diameter increases. The pores are connected to form cracks or crevices. The decline in compressive strength further substantiates this phenomenon observed with an increase in FTC in the late stage of the FTC, as depicted in Figure 5. This decline offers qualitative and quantitative insights into the alterations in the internal spatial morphology of DSC. From a macroscopic perspective, the augmentation of pore saturation and water saturation results in heightened water absorption in DSC. Conversely, a modest amount of pore absorption is conducive to culminating the hydration reaction in the subsequent stage. It is crucial to recognize that excessive water absorption and the FTC may induce significant self-stress in the concrete. Similarly, the densification effect of external compressive stress on concrete in the early stage becomes one of the factors contributing to concrete damage in the later stage. Over time, this stress may induce volumetric expansion in the concrete, resulting in surface fissures and a reduction in apparent density, significantly impairing its mechanical qualities and durability.

### 3.5. MIP

**Figure 8:**
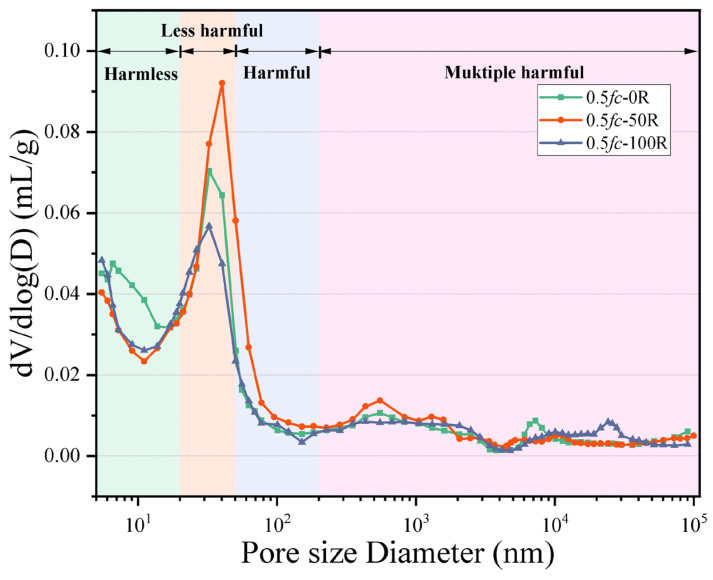
Pore size distribution.

The pore size distribution of desert sand concrete after 0, 50, and 150 freeze–thaw cycles at 0.5 *f*c is shown in Figure 8. Wu & Lian [42] classified the pores of concrete into four categories based on their diameters: those with pore diameters less than 20 nm are harmless pores, those with pore diameters between 20 nm and 50 nm are less harmful pores, those with pore diameters between 50 nm and 200 nm are harmful pores, and those with pore diameters more significant than the pore diameter of more than 200 nm are harmful pores. For the specimen with certain stress, with the increase in FTC times, the number of harmless pores with 50 times and 150 cycles is lower than the initial specimen. The number of less harmful pores with a pore size of 20 nm~50 nm is the most in the specimen with 50 times the FTC, which is because, with the FTC, the external water enters into the pore space of the cement paste and condenses and forms ice to make the pore space expand. After the melting of ice crystals, there is residual deformation in the form of pore shrinkage, which will gradually coarsen the pore structure of the cement paste. In this process, harmless pores will be expanded into less harmful pores, and the less harmful pores will be expanded into harmful pores. It is evident that the FTC exerts a gradual coarsening effect on the pore structure of the cement paste, with the coarsening effect generally occurring in pores with a diameter of 10 nm and above.

**Figure 9:**
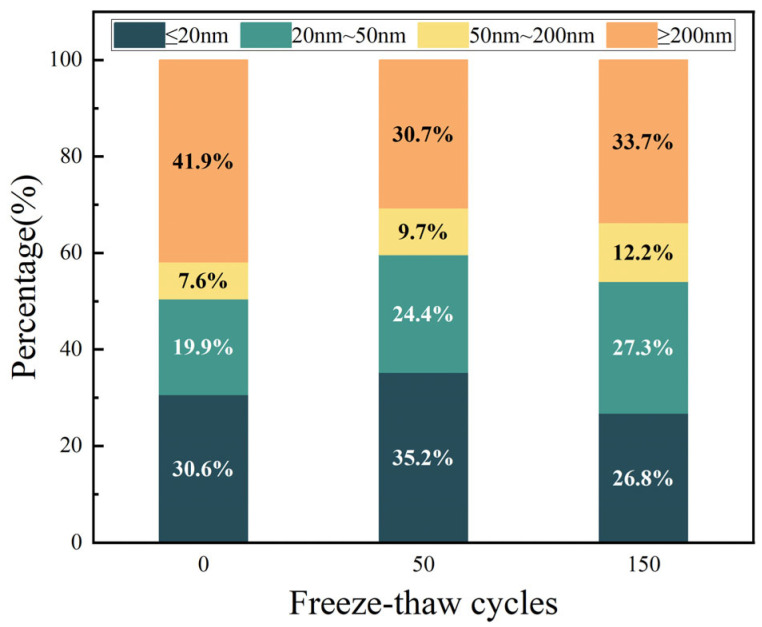
Percentage of pore types.

The results show that the percentage of non-harmful pores in concrete first increases and then decreases as the FTC progresses, the percentage of multi-harmful pores decreases and then increases with the FTC, and the percentage of less-harmful and harmful pores increases all the time with the FTC, as shown in Figure 9. This indicates that during the early stages of FTC, the hydration reaction continues. The external compressive stress, the generated C-S-H, occupies the larger pores in the concrete mortar so that the larger pores are filled with the corresponding pore diameter and become smaller, becoming a smaller class of pores, which well explains the reduction in hazardous pores in the early stages of FTC and the increase in other pores in the test results. As the FTC continues, the external compressive stress that previously played a compacting role has a counteracting effect on the concrete. As the internal hydration products nucleate and develop, internal stress is generated to resist external stress. The external load and internal stress act directly on the concrete. In addition, the FTC causes the pores to continuously absorb water, which then freezes and thaws, placing extreme pressure on and causing serious damage to the concrete. Further elaboration on the outcomes of the macro-scale experiments demonstrates that DSC exhibits an initial suppression of deterioration during the early stages of FTC under 0.5 *fc* stress, whereas it accelerates deterioration in the later stages of these cycles. Therefore, in the later stages of the FTC, the proportion of harmless pores decreased, and the proportion of harmful pores (≥20 nm) increased.

### 3.6. X-CT Characterization

**Figure 10:**
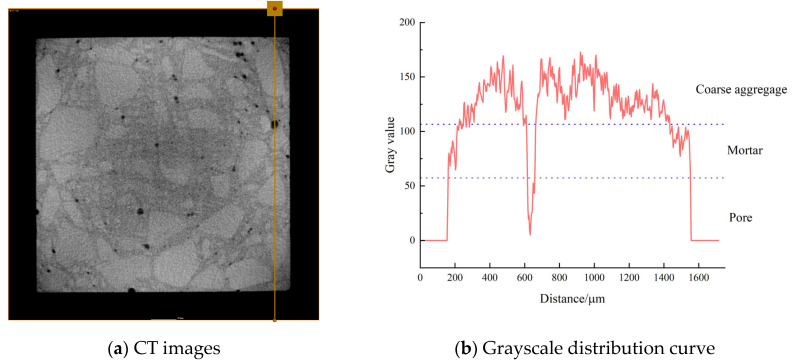
Grayscale division of each component of the CT image.

**Figure 11:**
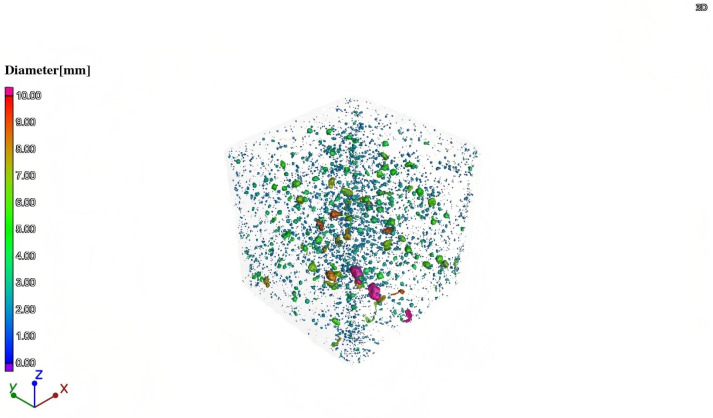
DSC three-dimensional pore distribution map.

CT scans were performed on DSC specimens subjected to 0.5 *fc* stress after undergoing 0, 50, and 150 FTC. The 2D slice images of the samples were obtained by scanning. The first step of image processing was noise reduction, and then the grey ranges of pores, mortar, and aggregate were differentiated according to the grey value. Figure 10a shows the drawing of a straight line in the two-dimensional slice image of the DSC through the mortar, aggregate, and pore position to draw a line segment. As illustrated in Figure 10b, the grey value of different phases in different subgroups varies, indicating that the range of grey values for each phase is distinct. Utilizing the grey values of 110 and 60, respectively, for the segmentation of mortar, coarse aggregate, and pore, it can be observed that when the grey value is less than 60, it can be identified as pores. Conversely, when the grey value ranges from 60 to 110, it can be identified as mortar, and values above 110 are indicative of coarse aggregate. Then, a three-dimensional reconstruction of member pores is carried out. Taking DSC subjected to a high-stress state as an example, Figure 11 shows the 3D model of pores after segmentation.

#### 3.6.1. Pore Sphericity

The sphericity of pores is a crucial metric for quantitatively defining the pore morphology of concrete. Its physical meaning is the ratio of pore volume to surface area. It is calculated as shown in Equation (5) [43].

(5) $$\psi =\frac{\sqrt[{3}]{\pi {\left(6V\right)}^{2}}}{A}$$

where V is the pore volume (mm^3^), and A is the pore surface area (mm^2^).

**Figure 12:**
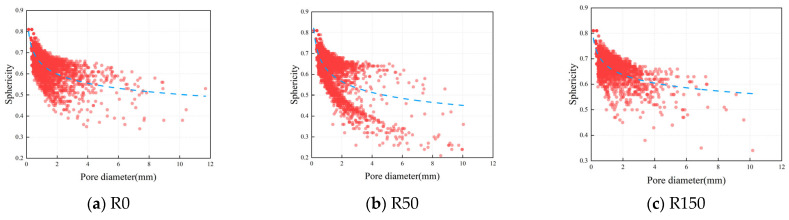
Pore scale versus sphericity.

The more the sphericity approaches 1, the more the pore form resembles a sphere. Poor sphericity of the pore structure may lead to localized stress concentrations at the pore margins, thus impacting the mechanical characteristics and durability of the concrete. To further examine the impact of FTC on the sphericity of pores in DSC under applied stress, the sphericity of pores in specimens subjected to 0, 50, and 150 FTC was assessed. Figure 12 illustrates a substantial negative association between pore sphericity and both pore volume and diameter, indicating that bigger pores exhibit less sphericity. This indicates that the larger the pore diameter, the greater the irregularity of the pores, which increases the stress concentration in the concrete. Consequently, the concrete is more susceptible to cracking and further deterioration due to this high-stress concentration.

**Figure 13:**
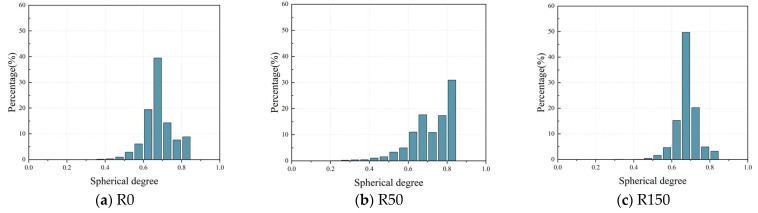
Frequency distribution of pore sphericity.

Figure 13 shows the sphericity frequency distribution of DSC after coupling high stress and FTC. The results show that the sphericity of the pores in DSC mainly has a unimodal distribution. The sphericity of the samples with 0 and 150 FTC is mainly concentrated between 0.6 and 0.7, accounting for 58.89% and 64.91% of the total sphericity, while the sample sphericity of the 50 FTC was concentrated at 0.8. The sample sphericity of the 0 and 150 FTC was mainly between 0.6 and 0.7, accounting for 58.89% and 64.91% of the total sphericity, respectively. Compared with the initial state, the sphericity of the specimens in the range of 0.6~0.7 and 0.7~0.8 for 150 cycles of freeze–thaw increased by 6.03% and 3.25%, respectively, while the proportion of sphericity in the range of 0.4~0.5 and 0.5~0.6 decreased by 0.71% and 2.83%, respectively. This suggests that freeze/thaw cycles reduce the proportion of small spherical porosity in the DSCs, resulting in more irregular porosity. The relationship between the pore size and the sphericity can explain this phenomenon—usually, the larger the sphericity, the smaller the diameter of the pore. Freeze–thaw damage typically manifests as a progressive enlargement of pore size, with pore morphology gradually shifting from regular to irregular shapes. This transformation is particularly pronounced in larger pores. Therefore, the larger the pores in the DSC under 0.5 *f*_c_ stress, the more susceptible they are to the effects of FTC, resulting in a decrease in the sphericity of the pores and a more irregular pore shape. The irregularity of this pore structure causes internal stress concentration, exacerbates damage to the fine-scale structure, and affects the overall durability and structural integrity of the concrete.

#### 3.6.2. Pore Shape Factor

To examine the impact of FTC on pore shape, the shape factor parameter was used to assess the pore morphology of concrete specimens before and after FTC. The shape factor is a measure of pore morphology determined by the ratio of elongation to flatness in three dimensions. Two pore morphology parameters, the flatness index (S/I) and the elongation index (I/L), were computed to quantitatively describe the three-dimensional pore morphology of DSC. The flatness index denotes the ratio of void thickness S to width I, whereas the elongation index signifies the ratio of void width I to length L. A ratio of 0.67 served as the threshold for classifying the pores in the concrete into cake, sphere, blade, and rod forms [44]. A greater ratio corresponds to a more complete pore morphology, while lower values of either or both indices indicate more flattened and elongated pores. Table 5 illustrates the classification of form factor indices.

**Figure 14:**
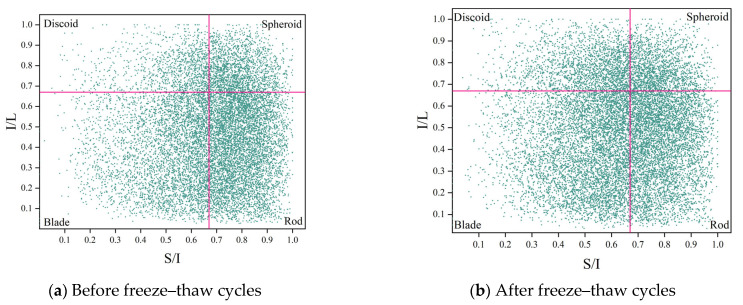
Zingg diagram of the distribution of pore form and shape.

The pore morphology of the DSC specimens was statistically analyzed using shape factor analysis in Figure 14. Table 6 shows the percentage of the four pore types in different specimens. It can be seen that at the initial state, the pore morphology in DSC is dominated by blade-like and rod-like pore; by the end of the FTC, the percentage of rod-like pores decreases by 11.08%, while cake-like pores increase by 11.97%. The possible reason is that the long axis of the rod-shaped pores is more extensive, so when water enters the interior of the concrete, these elongated pores quickly provide channels for water to diffuse. When the temperature decreases, the water in these pores freezes into ice. The pressure exerted by the ice on the pore walls causes the pores to expand, increasing the length of the short axis and making the pore shape more complete (more equant). Moreover, as the hydration reaction proceeds, it slightly increases the proportion of spherical pores and optimizes the pore morphology.

The X-CT findings of increased pore volumes and altered pore shapes after 150 FTC align closely with the MIP-detected shift toward larger pore diameters. Larger pores observed in the CT reconstructions are not only greater in volume but also exhibit lower sphericity (Figure 12), confirming they are more irregular in shape. Such irregular, expansive pores indicate that smaller pores have coalesced, leading to higher pore connectivity within the material. This enhanced connectivity, implied by the presence of larger (harmful) pores, facilitates easier water penetration and stress accumulation under freezing, thereby accelerating microstructural damage. The deterioration in pore morphology is further evidenced by the shape factor analysis (Figure 14 and Table 6), which shows a post-FTC increase in flattened (discoid) pores and a reduction in ideal spherical pores. These complementary observations from MIP and X-CT consistently highlight the same trend: progressive pore coarsening leads to an interconnected pore network with increasingly irregular pore geometry (i.e., a deterioration in pore shape), ultimately exacerbating stress concentrations and freeze–thaw deterioration.

### 3.7. Freeze–Thaw Damage Modelling and Life Prediction of Concrete Under Loading Based on Weibull Distribution

#### 3.7.1. Damage Model

Concrete is a multidirectional composite material, and its damage is randomly distributed in concrete, representing that freeze–thaw damage can be analyzed probabilistically. A two-parameter Weibull model is used for damage assessment with a probability density function of Equation (6):

(6) $$f\left(N\right)=\frac{\beta }{\alpha }{\left(\frac{N}{\alpha }\right)}^{\beta -1}\exp\left\{-{\left(\frac{N}{\alpha }\right)}^{\beta }\right\}$$

where α is a geometric parameter characterizing the material’s characteristic degradation lifespan, which correlates with concrete’s freeze–thaw resistance. A higher α indicates superior durability under freeze–thaw cycles. β is a shape parameter reflecting the randomness and uniformity of concrete’s freeze–thaw degradation process; a larger β signifies greater consistency in material properties and a more concentrated failure mechanism. Both α and β must satisfy α > 0 and β > 0.

Therefore, the damage probability distribution of concrete is formulated as Equation (7):

(7) $$F\left(N\right)=\int_{0}^{N}f\left(s\right)ds=1-\exp\left\{-{\left(\frac{N}{\alpha }\right)}^{\beta }\right\}$$

In this study, the damage variable D was defined based on the relative dynamic elastic modulus (RDEM), as it reflects the progressive degradation of the internal microstructure under freeze–thaw cycling:

(8) $$D_{E}=1-E_{r}=1-\frac{E_{n}}{E_{0}}$$

when N = 0, the damage probability is 0; when the damage value is reached, the damage probability is 1. Therefore, the concrete damage model is obtained by combining Equations (7) and (8).

(9) $$D_{E}=1-\exp\left\{-{\left(\frac{N}{\alpha }\right)}^{\beta }\right\}$$

The reliability function based on the probability of failure is shown in Equation (10):

(10) $$R\left(N\right)=1-F\left(N\right)=\exp\left\{-{\left(\frac{N}{\alpha }\right)}^{\beta }\right\}$$

The RDEM of desert sand concrete is evaluated as the deterioration index of its service life prediction under different freeze–thaw cycles, and the formula is shown in the following Equation (11):

(11) $$\omega_{2}=\frac{E_{r}-0.6}{0.4}$$

where ω_2_ is the RDEM evaluation parameter and *E*_r_ is the RDEM.

**Figure 15:**
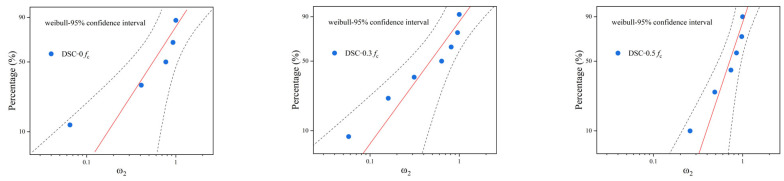
Weibull distribution test of deterioration indicators of desert sand concrete under different loads.

The deterioration index data of desert sand concrete under freeze–thaw cycles at different loads were subjected to a Weibull distribution test to verify whether they conform to the functional distribution. When the experimental data conform to the assumed distribution function at a significance level of 0.05, the data points tend to converge and are distributed near the diagonal line in the figure. The test results are shown in Figure 15.

As seen from Figure 15, the deterioration indexes of desert sand concrete under different dosage gradients are basically spread around the reference line on both sides, and all the points are discretely distributed within the 95 % confidence interval, so it can be determined that the functional assumptions are valid and the evaluation parameters of the RDEM obey the Weibull distribution [45].

The Weibull transform of the Equation (7) can be written in the following linear form:

(12) Y=A+BX

when *Y* = *ln*(−*ln*(1 − *F*(*N*))), *X* = *ln*(*N*), *B* = *β*, *A* = −*βlnα*.

**Figure 16:**
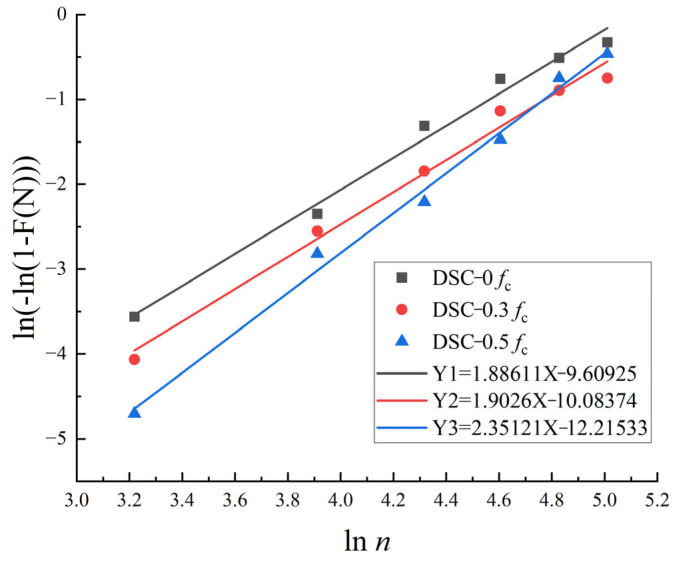
Durability life Weibull linear regression.

Weibull operations were performed by combining Equations (6)–(11), and Weibull analysis was performed to obtain the values of Weibull parameters A and B for DSC under different stress states, as shown in Table 7:

To obtain the correlation of the characteristic parameters, the damage level F(N) and the number of cycles N are substituted into Equation (12), and a least-squares fit is performed, as shown in Figure 16.

**Figure 17:**
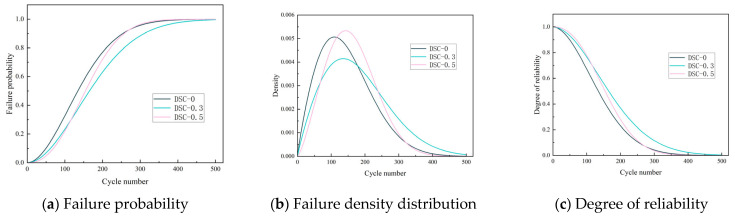
Failure probability, failure probability distribution, and reliability of concrete after freeze–thaw cycles.

The values of α and β have been determined. The derived values of α and β are incorporated into Equation (7) to calculate the failure probability of concrete for each condition during a single FTC, subsequently extended to 500 FTC. As illustrated in Figure 17a, the failure probabilities of specimens are similar under varying loads for FTC ranging from 0 to 50. However, the failure probability of specimens begins to rise markedly after 100 FTC, with DSC-0.5 exhibiting the most pronounced increase. The slope increases, indicating that the speed of the failure probability is accelerated, and the failure probability reaches more than 80% after 200 times FTC. In the specimens under medium horizontal stress, this phenomenon was significantly improved. Compared with the high-stress level and no stress level, the failure probability curves were lower than the latter in the middle and late stages of the FTC, which is because when there is a certain amount of applied load, it improves the overall durability of the concrete, and when there is an excessive amount of applied load in the early stage. However, the densification effect is more substantial, but it will lead to more serious damage in the later stage. In the DSC in the medium stress case, after 200, 300, 400, and 500 FTC, the probability of failure is reduced by 8.93%, 7.91%, 2.26%, and 0.34%, respectively, compared to the high-stress level.

Figure 17b shows the failure density function of the test specimen, which represents the macroscopic failure process of the material. As the curve peak increases and the span decreases, material degradation increases. Comparing the DSC specimens under different stress states, the sensitivity to FTC is still the largest for high stress, the second for no stress, and the smallest for medium stress, which also argues the previous analysis. Specimen reliability, which has a complementary relationship to specimen failure probability, is shown in Figure 17c. The higher the likelihood of the sample failing, the less reliable it is. Within 500 FTC, the reliability of all specimens decreases rapidly, with the fastest decrease in the DSC-0 group and the slowest decrease in the DSC-0.3 group, and the difference reaches a maximum of 27.86% at 199 FTC.

#### 3.7.2. Lifespan Prediction

The freeze–thaw conditions of concrete in outdoor environments vary from those in laboratory settings. Thus, indoor freeze–thaw measurements cannot clearly indicate the concrete’s service life. Li [46] analyzed various indoor and outdoor freeze–thaw situations and delineated the correlation between interior quick freeze–thaw and outside natural freeze–thaw, as shown in Equation (13).

(13) $$t_{s1}=\frac{K\cdot n}{M}$$

where t_s1_ represents the service life of concrete, K denotes the indoor–outdoor freeze–thaw ratio coefficient, n is the number of indoor freeze–thaw events, and M is the average number of outdoor freeze–thaw events per year.

In northwest China, it may appear in the annual FTC 118 times/year, different types of concrete with different construction conditions according to the current concrete test procedures set by the rapid freeze–thaw test method (similar to GB/T 50082-2024) indoor and outdoor comparative relationship between 1:10~1:15 between the large average of 1:12, that is, indoor, a rapid FTC is equivalent to the natural conditions of the 12 FTC.

If the relative modulus of elasticity is less than 60%, then the concrete is considered to be in a failure state if F(N) = 40%. By inserting the data of K = 12 and M = 112 into the damage progression equation of the corresponding sample block, it is possible to obtain the maximum number of FTC of each sample block, and the service life of the concrete under freeze–thaw conditions is obtained from Equation (13) and shown in Table 8.

The 12–15 years obtained herein represent a conservative lower-bound estimate under fully saturated conditions, rapid freeze–thaw acceleration, and an indoor–outdoor conversion ratio of K ≈ 12. In actual engineering practice, factors such as prolonged exposure to non-fully saturated conditions, smaller annual temperature ranges/shorter durations of extreme low temperatures, larger component dimensions (size effect), and the use of air-entraining/high-quality air-bubble systems can significantly reduce ice pressure and seepage-driven stresses, thereby extending durability life. Conversely, de-icing salts and high saturation–refreezing cycles may shorten service life, necessitating verification of conversion parameters based on environmental zoning. This study aligns with Wei et al.’s [47] prediction of approximately 20 years’ service life for concrete in northwestern environments. However, under optimal material mixes and milder exposure conditions, DSC systems may achieve significantly longer lifespans. The 12–15 years presented herein should therefore be understood as a conservative lower bound.

## 4. Conclusions

This article used custom equipment to investigate the frost resistance of the DSC under the simultaneous influence of continuous compression stress and FTC. The primary findings are as follows:

(1) Applying sustained loads to concrete mitigates crack propagation and surface paste spalling during freeze–thaw cycles to some extent. This occurs because the continuous loading counteracts part of the stress expansion caused by pore water freezing, thereby facilitating the nucleation and development of hydration products.
(2) The application of a compressive stress level of 0.3 *f*_c_ reduces the number of microcracks and large pores in the specimen, increases the complexity of the pore structure, and ultimately improves the freeze resistance of the DSC. An amount of 0.5 *f*_c_ is unfavourable to the DSC because it exacerbates the deterioration of the pore structure, which in turn reduces the freezing resistance of the DSC.
(3) The Weibull distribution model was used to ascertain the damage number and distribution parameter of the freeze–thaw guarantee number for the standard FTC. The probability link between freeze–thaw damage and the number of FTC for DSC in a freeze–thaw climate was further shown. A comprehensive examination of the damage curve for concrete in freeze–thaw conditions demonstrates a clear probability correlation between the number of FTC and the consequent damage. The paper also presents a thorough cumulative damage model that considers the impact of varying dependability levels on FTC. The study’s results provide a dependable forecast of the service life of concrete specimens depending on the quantity of interior and outdoor FTC. Moreover, the model’s dependability was rigorously validated, confirming the robustness and trustworthiness of the experimental findings. The study’s results provide a significant reference for analyzing damage patterns and predicting service life in freeze–thaw conditions.

Although this study has revealed the durability characteristics of desert sand concrete subjected to freeze–thaw cyclic loading, its limitations must be clarified. The evaluation of concrete specimens using relative dynamic modulus of elasticity as a deterioration indicator remains overly simplistic. Furthermore, the damage theories discussed herein are largely based on empirical derivations, lacking sufficient theoretical validation. Future research may incorporate computer vision-based methodologies to achieve automated quantitative assessment of surface damage and crack propagation in desert sand concrete under freeze–thaw conditions. For instance, Kabir et al. [48] employed deep convolutional neural networks for semantic segmentation, enabling precise identification of surface degradation zones within digital imagery. Concurrently, Song et al. [49] proposed a series of efficient convolutional architectures that strike a balance between accuracy and computational cost, proving particularly suitable for large-scale image analysis and crack propagation detection.

## Figures and Tables

**Table 1:** Main chemical composition of desert sand.

Ingredient	SiO_2_	Al_2_O_3_	CaO	Fe_2_O_3_	K_2_O	Na_2_O	MgO	TiO_2_	P_2_O_5_
Quantity	66.47	14.48	4.38	3.23	2.71	2.56	2.24	0.672	0.247

**Table 2:** Desert sand particle size distribution.

Particle size/mm	>0.16	0.075~0.16	<0.075
Cumulative sieve residual/%	11.8	88.2	10.2

**Table 3:** The main chemical composition is cement, fly ash, and steel slag powder/w%.

Ingredients	CaO	SiO_2_	Al_2_O_3_	Fe_2_O_3_	SO_3_	MgO	Na_2_O	K_2_O	Ti_2_O	MnO
Content	54.48	21.87	6.13	3.26	4.10	2.18	1.34	0.769	0.49	0.117
Fly ash	9.53	49.11	18.74	8.09	1.36	3.53	3.77	1.86	0.897	0.124
Steel slag	28.1	8.55	2.63	33.4	-	3.92	-	-	0.92	4.92

**Table 4:** Concrete mix ratio.

Type	Water (kg/m^3^)	Content (kg/m^3^)	Fly Ash (kg/m^3^)	Steel Slag (kg/m^3^)	Desert Sand (kg/m^3^)	Sand (kg/m^3^)	Aggregate (kg/m^3^)
DSC	155	241	69	34	257	599	1046

**Table 5:** Classification of shape factor indices.

Classification	Stretch Ratio	Flatness Ratio	Shape
I	>0.67	<0.67	Discoid
II	>0.67	>0.67	Spherical
III	<0.67	<0.67	Bladed
IV	<0.67	>0.67	Rod

**Table 6:** Proportion of four pore types before and after freeze–thaw cycles.

Sample No.	Discoid (%)	Spherical (%)	Bladed (%)	Rod (%)
Initial	12.26	15.19	32.16	40.39
Final	24.23	11.60	34.86	29.31

**Table 7:** Weibull linear regression results for durability life under freeze–thaw conditions.

Test Condition	A	B	α	β	R^2^
DSC-0	−9.6093	1.8861	163.1623	1.8861	0.9880
DSC-0.3	−10.0837	1.9026	200.3326	1.9026	0.9883
DSC-0.5	−12.2153	2.3512	180.4291	2.3512	0.9930

**Table 8:** Concrete service life based on climate in northwest China.

Test Condition	Limit Freeze–Thaw Cycles	Service Life (Year)
DSC-0	115	12.3
DSC-0.3	141	15.1
DSC-0.5	136	14.6

## Data Availability

The original contributions presented in this study are included in the article. Further inquiries can be directed to the corresponding author.
